# Association Between Myocardial Infarction and Triglyceride-Glucose Index: A Study Based on NHANES Database

**DOI:** 10.5334/gh.1303

**Published:** 2024-02-23

**Authors:** Dandan Zheng, Ligong Cao

**Affiliations:** 1 The Department of Cardiology, Xiangyang Central Hospital, Affiliated Hospital of Hubei University of Arts and Science, Xiangyang City, Hubei Province, China

**Keywords:** triglyceride-glucose index, myocardial infarction, NHANES, race, smoking, chronic obstructive pulmonary disease

## Abstract

**Objective:**

To investigate differences in levels of the triglyceride-glucose (TyG) index between individuals with myocardial infarction (MI) and those without MI, as well as the association between TyG index and risk of MI.

**Methods:**

Data from the National Health and Nutrition Examination Survey (NHANES) for US adults from 2013 to 2018 were included in this study. Using MI as an outcome variable and TyG index as an exposure variable, logistic regression models were employed to analyze relationship between MI and TyG index.

**Results:**

The study included 6,695 participants. Compared to the non-MI group, patients with MI had significantly higher TyG index (8.89 vs. 8.63, *P* = 0.003). Higher TyG index was significantly associated with an increased risk of MI in US adults (OR: 1.69, 95% CI: 1.26–2.26, *P* < 0.001). Race, smoking status, and history of chronic obstructive pulmonary disease (COPD) had significant impacts on the association between TyG index and risk of MI (*P* for interaction < 0.05). Subgroup analysis demonstrated a significant positive correlation between TyG index and MI risk in non-Hispanic Black individuals, non-smokers, and individuals without COPD across multiple models (OR > 1.0, *P* < 0.05).

**Conclusion:**

US adults with higher TyG index were more susceptible to MI, and TyG index may be used to identify individuals at high risk of MI in the US population.

## 1. Introduction

A major factor in death worldwide is cardiovascular disease (CVD) [1]. With 18.4 million fatalities in 2019, CVD estimated to account for 1/3 of all global deaths [2]. Myocardial infarction (MI) is one of the most severe and fatal forms of CVD [3]. Pathologically, MI is defined as myocardial cell death brought on by persistent and severe ischemia [4] and is a major manifestation of coronary artery disease (CAD). Myocardial infarction often leads to complications such as cardiac rupture [5], heart failure [6], cardiogenic shock [7], and malignant arrhythmias [8], which further worsen the prognosis of patients. Regardless of whether it occurs in high-income countries [9] or low- to middle-income countries [10], MI imposes a significant disease burden. Furthermore, the impact of MI on healthcare systems should not be underestimated. It has been reported that in 2010 the direct medical costs associated with hospitalization for MI in the US amounted to around $450 billion, and this figure is projected to rise to $1 trillion annually by 2030 [11]. Due to its acute onset, high mortality rate, poor prognosis, and high medical costs, MI has become a serious public health issue. Therefore, exploring the mechanisms of MI development and identifying risk factors is of great significance for its prevention, diagnosis, and treatment.

Modifiable risk factors, or those that may be decreased or managed by behavioral changes, are mostly responsible for the risk of MI [12], These variables include smoking, physical inactivity, poor food choices, high blood pressure, and obesity [13]. Metabolic factors, such as blood lipids, blood pressure, diabetes, and obesity [14], are consistently identified as major risk factors for CVD-related mortality, including MI. The development of biomarkers that can accurately identify high-risk individuals or susceptible populations for MI is therefore urgently needed. Triglyceride-glucose (TyG) index has emerged as a promising biomarker among various candidates due to its significant correlations with severity of CAD and long-term cardiovascular risk [15,16]. Fasting triglyceride and fasting glucose levels are combined to create the composite index known as the TyG index [17]. Although initially considered a biomarker for assessing insulin resistance (IR) [18], TyG index may be an independent predictor of CVD [19]. In patients with acute MI (AMI), a high TyG index often indicates the occurrence of heart failure and increased mortality risk [20]. Even in non-diabetic populations, for those with acute ST-segment elevation MI, a high TyG index is linked to a dismal prognosis [17]. Currently, prognostic value of TyG index in MI patients has been extensively studied in population-based prospective cohorts, but its value in relation to MI risk in the general population remains elusive.

To address this research gap, we aimed to evaluate the relationship between the TyG index and risk of MI by studying data from 6,695 US adults who participated in the National Health and Nutrition Examination Survey (NHANES) from 2013 to 2018. This research aimed to offer a theoretical framework for the prevention of MI in the general population.

## 2. Methods

### 2.1 Study population

The National Health and Nutrition Examination Survey database is a large, nationally representative, multi-stage, stratified study conducted on the US population. To evaluate nutrition and health of the US population, National Center for Health Statistics (NCHS), a division of the Centers for Disease Control and Prevention, conducts this survey [21,22]. The NCHS Institutional Review Board gave its approval to this cross-sectional survey, which collects information on demographics, food habits, physical examinations, laboratory testing, and questionnaires [23]. All participants gave their written, informed consent. The NHANES survey data are publicly available online for use by researchers and other users.^1^

**Figure 1:**
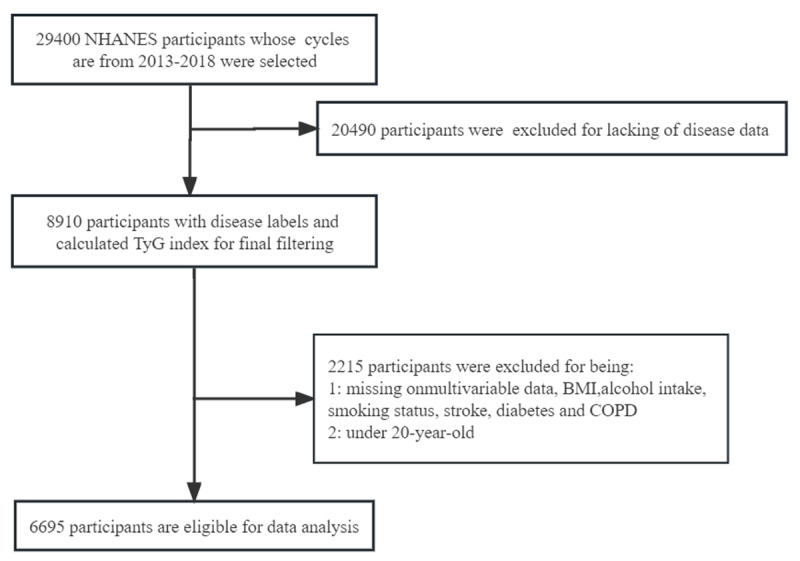
Flowchart of participant screening.

A total of 29,400 individuals participated in the NHANES study from 2013 to 2018. Participants with the following characteristics were excluded: (1) Missing data on serum glucose or triglyceride levels (n = 20,490); (2) Missing data on BMI, alcohol consumption, smoking status, stroke, diabetes, chronic obstructive pulmonary disease (COPD), and age <20 years (n = 2,215). Finally, our study included 6,695 participants, as shown in Figure 1.

### 2.2 Outcome variable: MI

We determined the presence of MI in participants using a medical conditions questionnaire. Participants who answered ‘yes’ to the question ‘Has a doctor or other health professional ever told {you/SP}that{you/s/he}had a heart attack (also called myocardial infarction (my-o-car- dee-al in-fark-shun)?’ were considered to have MI [3].

### 2.3 Exposure variable: TyG Index and related parameters

Triglyceride-glucose index is computed as the logarithm of the product of fasting triglyceride and fasting glucose, using the formula ln[triglyceride (mg/dL) × fasting glucose (mg/dL)/2] [24,25]. In NHANES, triglyceride levels were determined using the timed endpoint method with the DxC800, and fasting glucose was measured using the Roche Cobas C311 or Roche/Hitachi Cobas C501 chemistry analyzer with a hexokinase-mediated reaction.

Triglyceride-glucose index related parameters were calculated as follows [26]:

1. TyG-BMI = TyG index × BMI
2. TyG-WC = TyG index × WC
3. TyG-WHpR = TyG index × WHpR
4. TyG-WHtR = TyG index × WHtR

Body Mass Index, BMI; Waist circumference, WC; Waist-to-hip ratio, WHpR; Waist-to-height ratio, WHtR.

### 2.4 Covariates

Potential confounding factors were included as covariates in this study, including demographic information and self-reported medical history and health-associated behaviors. The demographic variables consisted of age (20–45, 45–69, ≥69), gender (male/female), and race (non-Hispanic White, non-Hispanic Black, Mexican American, other Hispanic, and other race) [27]. Weight (kg) divided by height squared (m^2^) was used to compute BMI, which was then split into ranges of ≤25, 25–30, and >30, respectively [28]. Self-reported medical history included a history of stroke, diabetes, and COPD. Stroke was defined as a positive response (‘yes’) to the question ‘Has a doctor or other health professional ever told you had a stroke?’ [29]. Diabetes was defined by any of the criteria: (1) being informed by a doctor of having diabetes, (2) taking antidiabetic medication, (3) HbA1c >6.5%, or (4) fasting plasma glucose level >126 mg/dL [30]. Chronic obstructive pulmonary disease was defined as a positive response (‘yes’) to the question ‘Ever told you had COPD?’ [31]. Health-associated behaviors included alcohol intake (yes/no) and smoking. Alcohol intake was defined as a positive response (‘yes’) to either ‘Had at least 12 alcohol drinks/1 year?’ or ‘Ever had a drink of any kind of alcohol’. Smoking status was categorized following question ‘Do you now smoke cigarettes?’ as ‘current smoker’ for those who answered ‘Every day’ or ‘Some days’, ‘former smoker’ for those who answered ‘yes’ to ‘Smoked at least 100 cigarettes in life’, and ‘never smoker’ for the remaining respondents [32].^2^

### 2.5 Statistical analysis

A baseline table was generated using the ‘tableone’ package, grouping the samples based on the presence of MI. The table provided sample sizes and proportions for categorical variables, and mean values and standard deviations for continuous variables (n was unweighted; n(%), mean, and SD were adjusted for weights). Weighted logistic regression models for MI and TyG indices were constructed using the ‘survey’ package, and the *P*-value of the interaction term in the stratified weighted logistic regression adjusted for confounding factors was tested using the chi-square test. Stratified on the basis of race, smoking status, and COPD history, weighted logistic models were constructed using the ‘survey’ package for MI and TyG indexes, respectively. The models included: Crude (unadjusted); Model 1 (adjusted for gender and age); Model 2 (adjusted for gender, age, BMI, smoking, and alcohol intake); Model 3 (adjusted for all confounding factors, including history of stroke, diabetes, and COPD). All data analyses were performed on R software (v 4.2.1), and *P* < 0.05 was considered statistically significant.

## 3. Results

### 3.1 Baseline characteristics

Among 6,695 participants, 51.3% were female, 88.0% were under the age of 69, 65.1% were non-Hispanic White, 82.0% had a history of alcohol intake, and 55.4% were never smokers. In terms of medical history, most patients did not have a history of diabetes (84.5%), stroke (97.0%), or COPD (96.0%). Overweight or obese individuals accounted for 71.5% of the samples, with an average TyG index value of 8.64. Baseline characteristics of all eligible participants from the NHANES dataset between 2013 and 2018 could be found in Table 1.

**Table 1:** Baseline Characteristics of Participants from 2013 to 2018.


CHARACTERISTICS	N(%)/MEAN ± SD

**Overall**	6695

**Gender (%)**	

Female	3430 (51.3)

Male	3265(48.7)

**Age (%)**	

20–45	2734 (45.9)

45–69	2824 (42.1)

≥69	1137 (11.9)

**Race (%)**	

Mexican American	983 (8.9)

Other Hispanic	720 (6.4)

Non-Hispanic White	2604 (65.1)

Non-Hispanic Black	1366 (10.9)

Other race	1022 (8.7)

**Alcohol intake(%)**	

No	1533 (18.0)

Yes	5162 (82.0)

**Smoke (%)**	

Never smoked	3728 (55.4)

Former smoker	1659 (26.4)

Current smoker	1308 (18.3)

**BMI (%)**	

≤25	1901 (28.4)

25–30	2171 (32.1)

>30	2623 (39.4)

**Diabetes (%)**	

No	5308 (84.5)

Yes	1387 (15.5)

**Stroke (%)**	

No	6433 (97.0)

Yes	262 (3.0)

**COPD (%)**	

No	6405 (96.0)

Yes	290 (4.0)

**TyG index (mean (SD))**	8.64 (0.66)

**Myocardial Infarct (%)**	

No	6384 (96.4)

Yes	311 (3.6)


*Note:* Categorical variables are presented as n (%), and continuous variables are presented as mean (sd); n is unweighted, while n (%), mean, and sd are weighted.

Based on the presence of MI as the classification criterion, a feature analysis was conducted (Table 2). The results showed a prevalence of 3.6% for MI. Age, smoking status, gender, and medical history (including diabetes, stroke, and COPD) were all shown to be significantly different between the two groups (*P* < 0.05). Among MI patients, the majority were male (64.7%), half of the patients (56.5%) were aged between 45 and 69, and 21.5% had a history of COPD. Furthermore, compared to non-MI individuals, MI patients had significantly higher TyG index values (8.89 vs. 8.63, *P* < 0.05).

**Table 2:** Characteristics of the Population by MI Groups.


CHARACTERISTICS	MYOCARDIAL INFARCT (NO)	MYOCARDIAL INFARCT (YES)	*P*-VALUE

**Overall**	6384	311	

**Gender (%)**			<0.001

Female	3315 (51.9)	115 (35.3)	

Male	3069 (48.1)	196 (64.7)	

**Age (%)**			<0.001

20–45	2710 (47.4)	24 (7.6)	

45–69	2673 (41.6)	151 (56.5)	

≥69	1001 (11.0)	136 (35.8)	

**Race (%)**			0.174

Mexican American	950 (9.1)	33 (4.4)	

Other Hispanic	688 (6.4)	32 (5.0)	

Non-Hispanic White	2449 (64.9)	155 (70.6)	

Non-Hispanic Black	1299 (10.9)	67 (11.5)	

Other race	998 (8.7)	24 (8.4)	

**Alcohol intake (%)**			0.877

No	1473 (18.1)	60 (17.6)	

Yes	4911 (81.9)	251 (82.4)	

**Smoke (%)**			<0.001

Never smoked	3624 (56.2)	104 (33.3)	

Former smoker	1530 (25.8)	129 (41.8)	

Current smoker	1230 (18.0)	78 (24.8)	

**BMI (%)**			0.511

≤25	1825 (28.5)	76 (25.2)	

25–30	2067 (32.0)	104 (35.6)	

>30	2492 (39.4)	131 (39.2)	

**Diabetes (%)**			<0.001

No	5147 (85.5)	161 (56.9)	

Yes	1237 (14.5)	150 (43.1)	

**Stroke (%)**			<0.001

No	6169 (97.5)	264 (84.5)	

Yes	215 (2.5)	47 (15.5)	

**COPD (%)**			<0.001

No	6154 (96.7)	251 (78.5)	

Yes	230 (3.3)	60 (21.5)	

**TyG index (mean (SD))**	8.63 (0.66)	8.89 (0.71)	0.003


*Note:* Categorical variables are presented as n (%), and continuous variables are presented as mean (sd); n is unweighted, while n (%), mean, and sd are weighted.

### 3.2 Association between TyG Index and MI

We used logistic regression to investigate association between the TyG index and MI (Table 3) and revealed TyG index as a risk factor for MI. A higher TyG index suggested an increased risk of MI (OR: 1.69, *P* < 0.001).

**Table 3:** Logistic Regression Analysis of TyG Index and MI.


CHARACTERISTIC	OR	95%CI	*P*-VALUE

**TyG index**	1.69	(1.26, 2.26)	**<0.001**


*Note:* No adjustment for any confounding factors.

### 3.3 Subgroup analysis

To investigate the variables impacting the relationship between the TyG index and MI risk, subgroup analysis was carried out. As presented in Table 4, race, smoking status, and COPD history had significant interactions with the association between TyG index and MI risk (*P* for interaction < 0.05). Therefore, logistic regression analysis was conducted stratified by race, smoking status, and COPD history (Table 5). It was found that among other Hispanic individuals (OR = 1.33, *P* = 0.021) and non-Hispanic White individuals (OR = 1.87, *P* < 0.001), TyG index was positively associated with MI risk only in the crude model (unadjusted for confounding factors). On the other hand, among non-Hispanic Black individuals (OR > 1.0, *P* < 0.05) and those without COPD (OR > 1.0, *P* < 0.05), the positive correlation between TyG index and MI risk was observed regardless of adjustment for confounding factors (both in crude and model I, II). In the never-smoker group (OR > 1.0, *P* < 0.05), the positive correlation between TyG index and MI risk was present in all models (crude and model I, II, III) (Table 5). Subsequently, we further investigated the relationship between the TyG Index and related indexes (obesity indexes: BMI, WC, WHtR, and WHpR) with MI. The data results revealed that, except for TyG-BMI, the indexes of TyG-related parameters in MI patients were significantly higher than those in non-MI patients (*P* < 0.05) (Supplementary Table 1). Logistic regression analysis of TyG-related indexes demonstrated a positive correlation with the risk of MI (OR > 1.0, *P* < 0.05) (Supplementary Table 2), particularly evident in the cases of TyG-WHtR and TyG-WHpR.

**Table 4:** Linear Regression of Interaction Terms and TyG Index.


PARTICIPANTS	OR	95%CI	*P*-VALUE	*P* FOR INTERACTION

**Gender**				0.055

Female	0.86	0.50, 1.47	0.6	

Male	1.28	0.79, 2.08	0.3	

**Age**				0.950

20–45	0.73	0.30, 1.77	0.5	

45–69	1.05	0.59, 1.86	0.9	

≥69	1.3	0.79, 2.15	0.3	

**Race**				**0.038**

Mexican American	0.73	0.30, 1.78	0.5	

Other Hispanic	0.61	0.34, 1.07	0.073	

Non-Hispanic White	1.28	0.79, 2.08	0.3	

Non-Hispanic Black	1.52	0.85, 2.73	0.14	

Other race	0.41	0.10, 1.72	0.2	

**BMI**				0.491

≤25	0.9	0.48, 1.71	0.7	

25–30	1.09	0.61, 1.93	0.8	

>30	1.3	0.81, 2.09	0.3	

**Smoke**				**0.029**

Never smoked	1.63	1.11, 2.40	0.01	

Former smoker	1.05	0.64, 1.73	0.8	

Current smoker	0.73	0.36, 1.46	0.4	

**Alcohol intake**				0.349

yes	1.24	0.79, 1.96	0.3	

no	0.7	0.42, 1.15	0.14	

**Diabetes**				0.870

no	1.18	0.70, 2.01	0.5	

yes	1	0.67, 1.50	>0.9	

**COPD**				**0.005**

no	1.27	0.86, 1.87	0.2	

yes	0.63	0.26, 1.53	0.3	

**Stroke**				0.580

no	1.13	0.75, 1.69	0.5	

yes	1.04	0.39, 2.76	>0.9	


*Note:* No adjustment for any confounding factors.

**Table 5:** Four Types of Linear Regression Models Adjusted for Confounding Factors.


PARTICIPANTS	MODELS	OR	95% CI	*P*-VALUE

**All participants**	Crude	1.69	1.26, 2.26	<0.001

	model I	1.42	0.98, 2.05	0.055

	model II	1.41	0.96, 2.08	0.067

	model III	1.12	0.76, 1.65	0.6

**Race**				

Mexican-American	Crude	1.37	0.80, 2.35	0.2

	model I	1.07	0.53, 2.15	0.8

	model II	1	0.39, 2.57	>0.9

	model III	0.73	0.30, 1.78	0.5

Other Hispanic	Crude	1.33	1.04, 1.71	0.021

	model I	1.12	0.83, 1.52	0.4

	model II	0.98	0.62, 1.57	>0.9

	model III	0.61	0.34, 1.07	0.073

Non-Hispanic White	Crude	1.87	1.29, 2.72	<0.001

	model I	1.52	0.96, 2.41	0.063

	model II	1.57	0.98, 2.53	0.052

	model III	1.28	0.79, 2.08	0.3

Non-Hispanic Black	Crude	2.34	1.53, 3.58	<0.001

	model I	2	1.28, 3.13	0.002

	model II	1.97	1.19, 3.29	0.006

	model III	1.52	0.85, 2.73	0.14

Other race	Crude	0.79	0.36, 1.71	0.5

	model I	0.55	0.19, 1.63	0.3

	model II	0.56	0.16, 1.99	0.4

	model III	0.41	0.10, 1.72	0.2

**Smoke**				

Never smoked	Crude	2.51	1.82, 3.45	<0.001

	model I	2.14	1.44, 3.18	<0.001

	model II	2.17	1.51, 3.12	<0.001

	model III	1.63	1.11, 2.40	0.01

Former smoker	Crude	1.39	0.98, 1.95	0.054

	model I	1.23	0.81, 1.86	0.3

	model II	1.2	0.77, 1.87	0.4

	model III	1.05	0.64, 1.73	0.8

Current smoker	Crude	0.96	0.45, 2.05	>0.9

	model I	0.82	0.37, 1.80	0.6

	model II	0.92	0.43, 1.94	0.8

	model III	0.73	0.36, 1.46	0.4

**COPD**				

No	Crude	1.93	1.47, 2.52	<0.001

	model I	1.61	1.15, 2.26	0.004

	model II	1.59	1.12, 2.26	0.007

	model III	1.27	0.86, 1.87	0.2

Yes	Crude	0.59	0.26, 1.35	0.2

	model I	0.64	0.29, 1.38	0.2

	model II	0.76	0.32, 1.81	0.5

	model III	0.63	0.26, 1.53	0.3


*Note:* Crude model: no adjustment; Model 1: adjusted for age and gender; Model 2: adjusted for age, gender, BMI, smoking, and alcohol intake; Model 3: adjusted for all confounding factors; Models in all participants groups include race.

## 4. Discussion

This study is a large-scale cross-sectional study conducted among US adults. Our findings demonstrated a positive correlation between TyG index and MI risk, indicating that higher TyG index values were implicated in an increased risk of MI. The association was also influenced by factors such as race, smoking history, and COPD history, with a significant positive correlation observed in non-Hispanic Black individuals, never smokers, and those without COPD. Simultaneously, combining the TyG Index with various obesity indexes, including BMI, WC, WHtR, and WHpR, exhibited a significant increase in MI patients. Logistic regression analysis indicated a positive correlation between TyG-related parameters and the risk of MI.

Previous studies have demonstrated that among high-risk groups for CVD, a high TyG index is linked to higher all-cause mortality and cardiovascular mortality [33]. Patients with higher TyG index values have a poorer prognosis due to an increased likelihood of CAD and more severe coronary artery lesions [34]. Several studies have reported the association between the TyG Index and arterial stiffness with respect to gender. Lee *et al*. [35] found that the TyG Index is independently associated with increased arterial stiffness. Furthermore, the odds ratio (OR) for elevated brachial-ankle pulse wave velocity (baPWV) in the highest and lowest quartiles of TyG Index (>75th percentile) was 2.92 in males and 1.84 in females. Another study from the China H-type Hypertension Registry demonstrated a positive correlation between the TyG Index and increased baPWV in hypertensive patients, with the impact of TyG Index elevation on the risk of baPWV increase being greater in males than females [36]. However, Nakagomi et al.’s study contradicted these findings, revealing an association between TyG Index and baPWV increase, with the correlation being stronger in females than males [37]. Contradictory results may be attributed to selection bias in the patient population. Among all CAD manifestations, MI is the most severe clinically [38]. Our study indicated that the TyG Index was indeed significantly higher in MI patients compared to the non-MI population. The relationship between TyG Index and the risk of MI has been previously reported in prospective studies. A community-based prospective cohort study spanning 11 years with a large sample size (n = 98849 cases) revealed that both baseline and long-term elevation of TyG Index levels were associated with an increased risk of MI. The study suggested that the TyG Index helped identify individuals at high risk of MI. Furthermore, the research demonstrated that the impact of high TyG Index on the risk of MI was more pronounced in females compared to males [39].

The TyG Index has emerged as an attractive choice due to the high accessibility of the biomarkers required for its calculation, namely, fasting plasma glucose (FPG) and triglycerides (TG) [40,41]. Functioning as a highly sensitive and specific biomarker for identifying IR, the TyG Index has been demonstrated to possess better predictive value than conventional parameters [42,43,44]. A recent systematic review, evaluating the TyG Index as a substitute for biochemical markers for IR, presented low to moderate quality evidence compared to the gold standards of Homeostatic Model Assessment of Insulin Resistance (HOMA-IR) and hyperinsulinemic-euglycemic clamp (HIEC) [18]. Insulin resistance refers to a diminished response of tissues to insulin stimulation and is considered a key factor in glucose and lipid metabolism [45]. It is commonly associated with metabolic abnormalities, such as hypertension, dyslipidemia, obesity, which are known precursors to cardiovascular diseases [46].

Insulin resistance may cause metabolic abnormalities that encourage the onset of CAD even in healthy people. According to research by Ding *et al*. [47], a higher TyG index in individuals without CAD can act as a standalone predictor of elevated future risk of CAD. Moreover, glucose metabolism imbalance caused by IR may result in permanent hyperglycemia, leading to increased inflammation and oxidative stress, which can cause cellular damage [48,49,50]. Myocardial IR can impair the heart through at least three mechanisms: changes in signal transduction, poor substrate metabolism control, and altered substrate transport [51]. For example, under normal physiological circumstances, insulin causes vasodilation by mediating the production of nitric oxide; but, when insulin signaling is disrupted, it lowers the bioavailability of nitric oxide and causes vascular stiffness [52]. Therefore, individuals with higher TyG index values are more prone to CAD. A high TyG index enhanced the MI risks, according to our study. Furthermore, studies focusing on critically ill CAD patients indicated correlations of higher TyG index with increased mortality rates and longer ICU stays, imposing a significant economic burden on families and society [53]. Given the current guidelines recommending 10-year CVD risk assessment for seemingly healthy adults [54,55], in conjunction with our study results and previous research, we highly advise using the baseline TyG index as a screening tool to assist identify those who are more likely to experience cardiovascular problems early on. Additionally, since TyG index combines fasting blood glucose and triglyceride levels, further research investigating targeted pharmacological interventions to improve prognosis is warranted.

Furthermore, BMI and WC are commonly used as simple, effective, and non-invasive anthropometric measures, serving as useful indicators for obesity and metabolic risk, primarily assessing overall body fat status [56]. However, they may not be ideal for measuring abdominal fat accumulation. WHpR and WHtR indexes are better suited to gauge central obesity (abdominal fat accumulation) [57,58,59]. Relevant studies have reported that the combination of the TyG Index with obesity indexes such as body weight and waist circumference may provide better predictions for IR, metabolic syndrome (MetS), and diabetes [26,56,60,61]. This study found that parameters combining the TyG Index with various obesity indexes, including BMI, WC, WHtR, and WHpR, were positively correlated with the risk of MI, particularly the TyG-WHtR and TyG-WHpR parameters. Therefore, future assessments of MI and cardiovascular diseases should involve large-scale studies that comprehensively consider the combined effects of TyG Index and the individual TyG Index. This will contribute to a more comprehensive understanding of the joint impact of TyG Index and obesity indexes on cardiovascular health in patients, providing a more robust basis for clinical assessments.

Additionally, we also revealed a positive correlation between the TyG index and the risk of MI in non-Hispanic White and Black individuals. Prior studies have reported racial differences in IR [62]. Reduced insulin sensitivity in non-Hispanic Whites has been associated with increased vascular function impairment and risk of heart disease [63], while the prevalence of IR in non-Hispanic Blacks is significantly higher than in Whites [64]. In a study on trends of cardiovascular risk factors in the US, non-Hispanic Blacks exhibited significantly higher 10-year atherosclerotic CVD risk compared to non-Hispanic Whites [65]. Among different populations, non-Hispanic Blacks have the highest incidence of CVD and are among the populations with the highest CVD mortality rates [66]. This disparity may be related to vascular dysfunction among different races, with non-Hispanic Blacks showing impaired vasodilatory capacity and exaggerated vasoconstrictive responses, possibly due to increased oxidative stress and reduced nitric oxide bioavailability [67]. The mechanisms underlying racial disparities in CVD risk remain to be fully elucidated, and future work in this direction is needed to fill the knowledge gap and enhance our understanding of CVD risk differences among different racial populations.

Growing evidence points to the importance of metabolism in chronic lung disorders. Elevated TyG index reflects blood lipid abnormalities and high blood glucose levels, which can impact lung structure and function [68,69]. Hyperinsulinemia and IR are common underlying pathologies of blood lipid abnormalities and diabetes, and they can induce bronchial hyperresponsiveness through changes in parasympathetic nerve signaling and promote subepithelial fibrosis [70,71]. A high TyG index is implicated in an increased risk of cough, wheezing, exertional dyspnea, and a diagnosis of chronic bronchitis [72]. Furthermore, the TyG index is a novel risk marker for future COPD events in women, with a positive correlation between TyG index and COPD risk [73]. Compared to individuals without COPD, patients with COPD are more susceptible to CVD, with higher incidence and hospitalization risks [74]. Acute exacerbations of COPD and declining lung function are implicated in increased CVD risk and mortality rates [75,76]. However, our study found a positive correlation between TyG index and MI risk in never-smokers and non-COPD population. This discrepancy may be related to the physiological and pathophysiological relationship between COPD patients and IR, with shared risk factors such as smoking, lack of physical activity, and systemic effects (inflammation, oxidative stress, etc.) [77]. Insulin resistance is common in COPD, and in this population, a higher TyG index may indicate poorer lung and airway symptoms rather than CVD risk in comparison to never smokers and non-COPD population. We recommend assessing insulin sensitivity in COPD patients to reduce the incidence of acute exacerbations and improve their health status. Analyzing the TyG index can assist identify those who are at risk for MI or other CVD events in the non-COPD population.

While our study revealed a positive correlation between TyG index and MI risk, it is important to note some limitations. First, our study was an observational study and failed to establish a causal relationship, warranting further prospective trials to confirm our findings. Second, while we attempted to incorporate as many confounding factors as possible, other potential factors influencing metabolism, such as dietary habits or energy intake, were not included in our analysis and may have an impact on the results. Lastly, the conclusions of this study may not apply to people beyond the US because it was done on US adults.

## Data Accessibility Statement

The datasets generated and analyzed during the current study are not publicly available but are available from the corresponding author on reasonable request.

## Additional File

The additional file for this article can be found as follows:

10.5334/gh.1303.s1 Supplementary File. Supplementary Tables 1 and 2.

## Footnotes

Please visit the internet page at (https://www.cdc.gov/nchs/nhanes/index.htm) for additional details about NHANES.

The website (www.cdc.gov/nchs/nhanes/) provides the measurement/questionnaire procedures for each variable.
