# Supplementary material for: Association Between Myocardial Infarction and Triglyceride-Glucose Index: A Study Based on NHANES Database
Source: Glob Heart. 2024 Feb 23;19(1):23. doi: 10.5334/gh.1303 (PMC10885828; doi:10.5334/gh.1303)
Supplement: Supplementary File. — Supplementary Tables 1 and 2. [file gh-19-1-1303-s1.pdf]

Supplement Table 1 Table of TyG and other parameter population characteristics under MI grouping

Characters	Myocardial Infarct (No)	Myocardial Infarct (Yes)	P-value
**TyG index (mean (SD))**	8.63 (0.66)	8.89 (0.71)	**0.003**
**N (%)**	6384 (96.4)	311 (3.6)	
**TyG-BMI (mean (SD))**	256.05 (71.22)	267.99 (77.26)	0.127
**N (%)**	6384 (96.4)	311 (3.6)	
**TyG-WC (mean (SD))**	870.85 (184.22)	933.19 (178.00)	**0.001**
**N (%)**	6224 (95.6)	289 (4.4)	
**TyG-WHtR (mean (SD))**	5.18 (1.09)	5.55 (1.00)	**0.001**
**N (%)**	6224 (95.6)	289 (4.4)	
**TyG-WHpR (mean (SD))**	8.09 (1.07)	9.04 (0.84)	**<0.001**
**N (%)**	2026 (96.6)	105 (3.4)	

Note. Hip circumference was only included in 2017-2018 cycle

Supplement Table 2 Logistic regression analysis of TyG and other parameters with MI

Participants	Models	OR	95% CI	P-value
**TyG-index**	Crude	1.69	1.26, 2.26	**<0.001**
	model I	1.42	0.98, 2.05	0.055
	model II	1.41	0.96, 2.08	0.067
	model III	1.12	0.76, 1.65	0.600
**TyG-BMI**	Crude	1.00	1.00, 1.00	0.090
	model I	1.00	1.00, 1.01	0.093
	model II	1.01	1.00, 1.01	**0.002**
	model III	1.00	1.00. 1.01	0.200
**TyG-WC**	Crude	1.00	1.00, 1.00	**<0.001**
	model I	1.00	1.00, 1.00	0.067
	model II	1.00	1.00, 1.00	**<0.001**
	model III	1.00	1.00,1.00	0.200
**TyG-WHtR**	Crude	1.33	1.15, 1.54	**<0.001**
	model I	1.23	1.02, 1.49	**0.024**
	model II	1.67	1.33, 2.09	**<0.001**
	model III	1.30	0.99, 1.71	**0.050**
**TyG-WHpR**	Crude	2.17	1.73, 2.68	**<0.001**
	model I	1.71	1.26, 2.34	**0.024**
	model II	1.93	1.18, 3.14	**<0.001**
	model III	1.67	1.26, 2.21	**<0.001**
